# Connections between ApoE, sleep, and A**β** and tau pathologies in Alzheimer’s disease

**DOI:** 10.1172/JCI171838

**Published:** 2023-07-17

**Authors:** Katherine R. Sadleir, Robert Vassar

**Affiliations:** 1 Davee Department of Neurology and; 2 Mesulam Center for Cognitive Neurology and Alzheimer’s Disease, Feinberg School of Medicine, Northwestern University, Chicago, Illinois, USA.

## Abstract

In this issue of the *JCI*, Wang and colleagues investigate the relationship between sleep disturbances, an environmental risk factor for Alzheimer’s disease (AD), and the apolipoprotein 4 (*APOE*ε*4*) allele, a strong genetic risk factor for AD. The authors subjected an amyloid mouse model expressing human APOE3 or APOE4, with and without human AD-tau injection, to sleep deprivation and observed that amyloid and tau pathologies were worsened in the presence of APOE4. Moreover, decreased microglial clustering and increased dystrophic neurites around plaques were observed in sleep-deprived APOE4 mice. In addition, aquaporin 4, important for clearing amyloid-β through the glymphatic system, was reduced and less polarized to astrocytic endfeet. These APOE4-induced changes caused alterations in sleep behavior during recovery from sleep deprivation, suggesting a feed-forward cycle of sleep disturbance and increased AD pathology that can further disrupt sleep in the presence of APOE4.

## Alzheimer’s disease risk factors

Alzheimer’s disease (AD) is the most common cause of dementia, affecting 6.7 million people in the United States alone (1). While therapeutic agents targeting the two main pathologies, extracellular amyloid-β (Aβ) plaques and intracellular tau neurofibrillary tangles, are in development, and three anti-amyloid antibodies have either been given FDA approval (aducanamab, lecanamab) or will be submitted for approval soon (donanemab), there is still a need to understand the risk factors for AD and their contribution to disease etiology. AD risk factors are both genetic and environmental, with probable interactions between them. In this issue of the *JCI*, Wang et al. (2) used AD mouse models to begin to understand the complex relationship between the strongest AD genetic risk factor, the apolipoprotein 4 (*APOE*ε*4*) allele, and an environmental factor, chronic sleep deprivation.

Sleep disturbances are common in patients with AD and can play a role in institutionalization (3), but actually begin before cognitive decline in the presymptomatic stage of AD (4). AD mouse models show that sleep deprivation increases Aβ deposition, whereas promoting sleep by blocking the orexin receptor can decrease amyloid pathology (5, 6). Sleep also affects tau: acute sleep deprivation increases extracellular tau, and chronic sleep deprivation increases the spread of AD-tau aggregates (7).

While sleep disruption has a clear worsening effect on both Aβ and tau pathologies, Wang and colleagues sought to understand the interaction between sleep deprivation and the main genetic risk factor for AD, the *APOE*ε*4* allele (2). The APOE genotype strongly affects AD risk, as individuals who carry APOE4 are substantially more likely to develop AD than are individuals with APOE3, the most common allele (8). APOE4 is also associated with increased risk of sleep disturbances such as obstructive sleep apnea (9), which causes intermittent hypoxia and disrupted sleep patterns. In addition, the effect of the *APOE*ε*4* allele on sleep disruption is associated with the severity of AD (10).

## Modeling APOE4 and sleep deprivation in mice

In the current study, Wang and colleagues used transgenic and knockin mouse lines to model the effects of the interaction between the APOE genotype and sleep deprivation on amyloid and tau pathologies (2). The murine Aβ peptide does not aggregate to form amyloid plaques, so the authors used the well-characterized APPPS1-21–transgenic mouse line (11) that expresses human amyloid precursor protein (APP) with the Swedish mutation (KM670/671NL) (12) and human presenilin 1 (PS1) with the L166P mutation (13), both of which cause early-onset autosomal dominant AD in humans. Since mouse and human APOE differ at the sequence and functional levels, the authors crossed APPPS1-21 mice with APOE-knockin lines, in which the murine APOE locus was engineered to encode human *APOE*ε*3* or *APOE*ε*4* alleles (14), referred to as APPPS1:E3 or APPPS1:E4, respectively. Since the APPPS1-21 mouse model does not have the fully developed tau pathology found in AD, tau seeding and spreading were induced by injecting sarkosyl-insoluble tau aggregates isolated from the postmortem brains of patients with AD (15–18) into the brains of APPPS1:E3 or APPPS1:E4 mice. This experimental approach allowed the authors to observe the effects of sleep deprivation in the context of APOE3 or APOE4 on amyloid and tau pathologies in the same mouse (2).

To model sleep deprivation, mice spent eight weeks in a specially designed cage in which a bar was programmed to move horizontally across the cage floor to wake the animals every 30 seconds during six hours of daylight, when mice normally sleep. The cages were also outfitted with piezoelectric sensors that detect pressure variations due to movement of the mice, so the authors could measure the length of the sleep bouts to determine how the APOE genotype and amyloid and tau pathologies interacted to affect recovery after sleep deprivation (2).

## Sleep deprivation has multiple effects in APOE4 mice

Wang and colleagues began their study by demonstrating that chronic sleep deprivation increased amyloid load as measured by the areas of Aβ immunostaining and the fibrillar amyloid–binding dye methoxy-X34 in the cortex, hippocampus, and thalamus of APPPS1:E4 mice compared with APPPS1:E3 mice. Since activated astrocytes and microglia cluster around plaques and phagocytose Aβ, the authors investigated how sleep deprivation in the context of the different *APOE* alleles affected glial activation. Glial fibrillary acidic protein–positive (GFAP-positive) astrocyte clustering around plaques was reduced in the cortex of APPPS1:E4 male mice after sleep deprivation, whereas it was increased in the thalamus of both male and female APPPS1:E4, but not APPPS1:E3, mice. Microglial clustering around plaques as measured by ionized calcium–binding adaptor molecule 1 (IBA1) positivity was decreased in all regions in APPPS1:E4 sleep-deprived mice compared with APPPS1:E3 mice (2).

The amount of microglial clustering around plaques is known to affect the formation of dystrophic neurites (19), which are swollen axons that surround amyloid plaques and accumulate stalled lysosomal and endosomal vesicles, autophagic intermediates, and the β-secretase enzyme BACE1. Again, sleep deprivation in the APPPS1:E4 mice increased BACE1-positive dystrophic neurites specifically in the hippocampus of females and in the thalamus of males, while sleep deprivation had no effect on dystrophic neurites in the APPPS1:E3 mice. From these data the authors conclude that sleep deprivation in the presence of Aβ deposition and APEO4 reduces the ability of microglia to phagocytose amyloid, thus promoting plaques that cause more severe neuritic damage (2).

Previous work shows that reduced microglial clustering and the resulting increase in dystrophic neurites (20, 21) can increase tau seeding and spreading in APP-transgenic mice injected with AD-tau (19, 22). To determine whether the increased neuritic dystrophy caused by sleep deprivation leads to increased tau spreading, the authors performed unilateral injection of AD-tau into the dentate gyrus and the overlying cortex of APPPS1:E4 and APPPS1:E3 mice, and then subjected these mice to sleep deprivation for 8 weeks. Labeling with the AT8 antibody, a marker of AD-tau pathology, showed increased staining in dystrophic neurites around plaques (NP-tau) on the ipsilateral cortex and increased tau spreading to the contralateral cortex in APPPS1:E4, but not APPPS1:E3, mice. Wang and colleagues further found that the increased NP-tau seeding and spreading in the APPPS1:E4 sleep-deprived mice was associated with decreased microglial clustering around plaques and increased BACE1-positive dystrophic neurites, supporting the link between dystrophic neurites and tau seeding and spreading (2).

## APOE4 and Aβ clearance through the glymphatic system

The APOE genotype is also known to affect clearance of Aβ from the brain, so the authors investigated the effects of APOE4 and sleep deprivation on Aβ glymphatic clearance mediated by the aquaporin 4 (AQP4) water channel. Normally, astrocytic AQP4 is localized in a polarized manner, concentrating at the astrocytic endfeet contacting the endothelial cells of the vasculature (23). Using confocal microscopy, Wang and colleagues found reduced polarization of AQP4 to blood vessels in sleep-deprived APPPS1:E4 mice compared with controls. Correspondingly, chronic sleep deprivation decreased the amount *Aqp4* mRNA only in the presence of APOE4. Expression of the homeostatic microglial genes *P2ry12* and *Tmem119* was also decreased, but there was no evidence of an increase in proinflammatory factors. Using a recently developed protocol (24), the authors separated the vascular compartment from the rest of the brain tissue and measured AQP4 levels by immunoblotting. AQP4 was enriched in the vascular compartment, indicating a physical association between perivascular astrocyte endfeet and endothelia, but AQP4 was reduced in APPPS1:E4 mice after sleep deprivation compared with APPPS1:E3 mice, indicating disruption of astrocytic AQP4 function (2).

## Increased pathology associated with APOE4 exacerbates sleep disruption

While sleep disruption exacerbates Aβ and tau pathologies, data also support the detrimental effect of AD pathology on sleep patterns. Since APOE4 increased Aβ and tau pathologies, the authors hypothesized that it would also change sleep behavior. In the first and final weeks of sleep deprivation, the authors measured sleep bout lengths during daylight hours right after sleep deprivation, as well as during nighttime sleep. They found that in the first week, daytime sleep bout lengths were increased in AD-tau–injected APPPS1:E4 mice compared with uninjected mice. Furthermore, during the final week, the AD-tau–injected APPPS1:E4 mice had a marked increase in the length of nighttime sleep bouts (when nocturnal mice are normally active) compared with controls. The authors suggest that the more severe Aβ and tau pathologies found in these animals resulted in greater damage to neurons regulating sleep-wake cycles, thus driving this abnormal shift from daytime to nighttime sleep (2).

## Microglia and astrocytes in APOE4-related sleep deprivation

**Figure 1:**
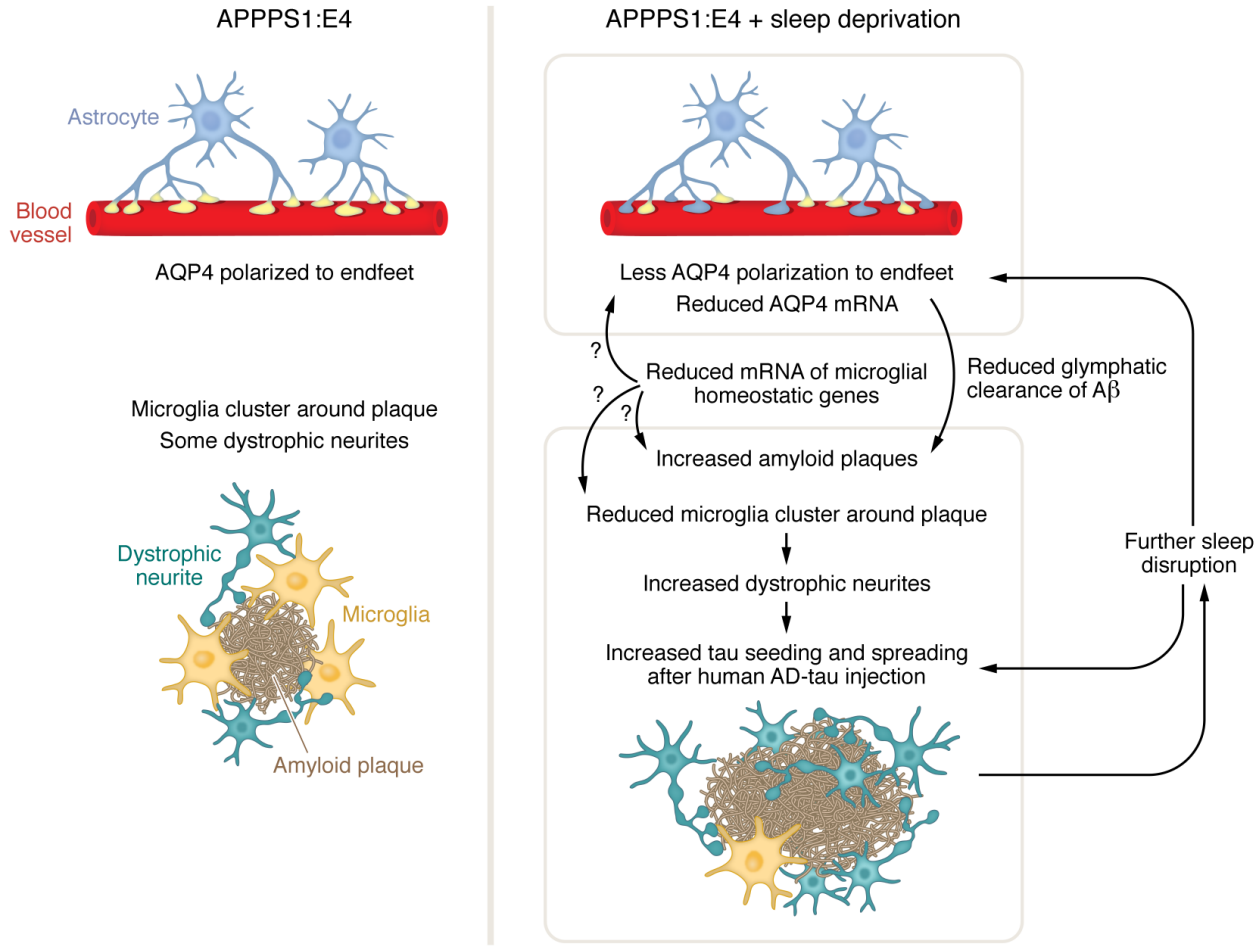
Sleep deprivation in the context of APOE4 results in a feed-forward loop that perpetuates neuropathological features and further disrupts sleep. The APPPS1:E4 mouse model develops pathology, including dystrophic neurites around amyloid plaques. Sleep deprivation in APPPS1:E4 mouse models results in microglial dysfunction that worsens amyloid and tau pathology, leading to further sleep disruption in a feed-forward loop. Structural changes include increases in the amyloid plaque area and fewer microglia clusters around plaques, which causes greater dystrophic neurite formation. In the presence of AD-tau, increased dystrophic neurites lead to further tau seeding and spreading. In addition, altered astrocyte function and reduced polarization of AQP4 to endfeet on blood vessels can impair glymphatic clearance of Aβ and lead to a higher plaque load. The expression of microglial homeostatic genes is reduced, which could further increase the plaque load, decrease microglial clustering, and induce astrocytic changes such as AQP4 reduction. APOE4 may also directly affect astrocyte function, as it is highly expressed in astrocytes.

The work of Wang and colleagues provides evidence that the effects of sleep deprivation on Aβ deposition and tau seeding and spreading and, conversely, the effects of AD pathology on sleep disruption are exacerbated by the presence of the *APOE-*ε*4* allele, suggesting a feed-forward cycle that may be more detrimental and harder to break in the context of APOE4 (Figure 1). The Wang et al. data suggest that two mechanisms could be involved, one microglial and the other astrocytic. First, in the context of sleep deprivation and APOE4, the microglial response to plaques is altered, with loss of microglial clustering around plaques and increased neuritic dystrophy. Consequently, tau seeding and spreading are enhanced with further disruption to sleep patterns, likely to due to worsening Aβ and tau pathologies. Second, glymphatic clearance of Aβ may be reduced by sleep deprivation in individuals carrying the *APOE*ε*4* allele as a result of changes in the levels and polarization of astrocytic AQP4, thus increasing amyloid load. The authors propose future work crossing APPPPS1:E4 mice, which have loxP sites flanking the knockin human *APOE* gene, with cell type–specific cre-driver lines to delete *APOE* only in microglia or astrocytes. This experiment would clarify the roles of microglia and astrocytes in the mechanisms that connect APOE4 and sleep deprivation to Aβ and tau pathologies in AD. It is also possible that microglial dysfunction lies upstream of astrocytic change, as microglia affect astrocyte activity in many ways. Finally, to translate the work of Wang and colleagues into therapeutic approaches for AD, further study assessing the effects of improved sleep in APOE4 carriers on AD risk or AD progression in preclinical or early stages of disease is necessary.

## Version 1 07/17/2023

Electronic publication
